# Investigating the differences in *Salmonella* serovar transmission within broiler production

**DOI:** 10.3389/fvets.2026.1812851

**Published:** 2026-04-17

**Authors:** David Ayala Velastegui, Nikki W. Shariat

**Affiliations:** 1 Department of Population Health, University of Georgia, Athens, GA, United States; 2 Center for Food Safety, University of Georgia, Griffin, GA, United States

**Keywords:** breeders, broilers, farm, *Salmonella*, vertical transmission, serovar

## Abstract

Almost 20% of human salmonellosis cases in the United States are linked to the consumption of undercooked, contaminated chicken. The prevalence of *Salmonella* in broiler breeder flocks is significantly lower than in broiler flocks, where typically 9 of 10 flocks test positive. Therefore, there is a need to understand how broiler flocks become colonized with *Salmonella*. This study aimed to assess the transmission of specific *Salmonella* serovars by profiling serovar populations at different stages of broiler production. In this study, we collected a total of 368 samples from 6 broiler complexes located across 4 states in the southeastern United States. Environmental samples were collected from breeder houses (*n* = 53), hatcheries (*n* = 45), and broiler houses immediately before chick placement (*n* = 44) and 7 days after placement (*n* = 45). These samples were analyzed to determine whether *Salmonella* found in broilers originates from earlier stages or from resident *Salmonella* persisting within the broiler house. Approximately one-quarter of pre-placement samples were *Salmonella-*positive, while, after 1 week, all broiler houses tested positive (Fisher’s Exact test, *p* < 0.05). Serovar-specific transmission dynamics were observed: Serovar Enteritidis originating from broiler breeders and the hatchery colonized new chicks, while residual serovar Infantis from previous broiler flocks was responsible for colonizing the newly placed chicks. Applying deep serotyping demonstrated that 7-day-old broiler flocks harbored more complex *Salmonella* serovar populations than those observed at the other three stages (Fisher’s, *p* < 0.01). Understanding the transmission differences among different serovars will facilitate the implementation of broad *Salmonella* control strategies and targeted interventions for specific serovars, ultimately improving poultry food safety.

## Introduction

*Salmonella enterica* is a leading bacterial foodborne pathogen in the United States, causing an estimated 1.3 million cases and 12,500 hospitalizations (1). *Salmonella* is composed of approximately 2,600 serovars, which are characterized by their somatic (O) and flagellar (H) antigens (2, 3). Despite this large number of serovars, it is estimated that 70% of salmonellosis cases are attributed to only 20 serovars (4). From 2019 to 2024, the most frequent serovars associated with human illness, according to the Centers for Disease Control and Prevention (CDC), were Enteritidis, Newport, and Typhimurium. Furthermore, serovars differ in their pathogenicity, virulence, and adaptation to different hosts (5, 6), highlighting the need to identify the serovars present in a particular food production system in order to assess the food safety risk associated with that product.

Between 2016 and 2022, the proportion of human salmonellosis cases linked to the consumption of contaminated, undercooked chicken increased from 12.7 to 19.7% in the United States (7, 8). Conversely, data from the United States Department of Agriculture-Food Safety and Inspection Service (USDA-FSIS) show that *Salmonella* prevalence in chicken parts decreased by more than half, from 18.5 to 7.4%, during the same time frame (9). As this reduction in prevalence at processing has not been accompanied by a reduction in the number of salmonellosis cases linked to chicken consumption, there is a need to better understand *Salmonella* dynamics throughout chicken production. While this disconnect may be explained by an increase in chicken consumption over this time frame (10), it may also reflect limitations of the USDA-FSIS performance standards, which, because they are based on *Salmonella* prevalence, do not account for differences in pathogenicity among different *Salmonella* serovars found in poultry, nor do they account for *Salmonella* quantity in the product. The most common serovars found in broilers at processing are serovars Kentucky, Infantis, Enteritidis, and Typhimurium (9, 11). Of these, serovar Kentucky is rarely associated with human illness in the United States (12–14) and is not considered highly pathogenic (15). The other three serovars rank among the top 10 serovars reported annually by the CDC (16).

In the United States, the broiler chicken industry is vertically integrated, where the multiple stages of production, from breeder pullets (sexually immature breeders) to processing plants, are contained within a single complex. This strategy allows improved and more efficient coordination across production stages and provides enhanced control over bird health management and food safety. A broiler integrator company can have multiple complexes, and food safety management practices (e.g., vaccination) are typically complex-dependent, based on the specific risk at a particular complex (e.g., the presence of a specific serovar) (17).

Within a complex, *Salmonella* can be transmitted vertically from broiler breeders (parents) to broilers (progeny, meat birds) (18–21). True vertical transmission occurs when the pathogen is transmitted within the egg, which requires ovarian colonization in the hen. Serovar Enteritidis is one of the few serovars that can be vertically transmitted in this manner (18, 22). As *Salmonella* colonizes the gastrointestinal tract of chickens, when the egg exits the cloaca, any *Salmonella* being excreted can contaminate the eggshell. Pseudo-vertical transmission occurs when a chick pips out during hatching, ingests *Salmonella,* and becomes colonized (23, 24). Horizontal transmission routes include contaminated feed, rodents, and beetles (17, 25–31), as well as transmission from colonized birds to naïve birds (19). Contaminated birds in a facility can cause the contamination of subsequent flocks raised in the same environment, resulting in potentially contaminated carcasses and posing a risk to human consumption (20, 32, 33).

Over time, different strategies have been implemented to reduce the incidence of *Salmonella* in poultry production. Research showing that identical *Salmonella* subtypes were present in broiler breeder flocks and on carcasses at processing (20) has led to a major focus on controlling *Salmonella* at the broiler breeder stage in commercial production over the past two decades. This has been achieved through enhanced on-farm biosecurity measures and the use of vaccinations to control specific serovars in broiler breeders (34). In the United States, broiler breeder vaccination programs include live-attenuated vaccines targeting *Salmonella* serovar Typhimurium (33). Killed vaccines are also used, typically as a commercial bacterin targeting serovar Enteritidis or as an autogenous vaccine containing two or three different serovars (or strains) (34). Only live-attenuated vaccines can be used in broiler flocks, and currently, the only commercial live-attenuated vaccines available in the United States are labeled to target serovar Typhimurium. A recent study found that serovars of great public health concern (Enteritidis, Typhimurium, and Infantis) accounted for only 6% of the overall prevalence in broiler breeders, compared to 81% for serovar Kentucky (17). Despite this significant success, likely due to increased *Salmonella* monitoring and vaccination in broiler breeders, serovars Enteritidis, Typhimurium, and Infantis still accounted for 42 and 48% of *Salmonella* recovered by the USDA-FSIS in processing establishments during the same period and location, specifically in broiler carcasses and parts, respectively (11). Furthermore, although only 33% of broiler breeders are colonized with *Salmonella,* 85% of broiler flocks test positive (17, 35). This higher prevalence, along with the increased occurrence of serovars of concern at processing, underscores the need to re-evaluate how broilers become colonized with *Salmonella.* Resolving this issue will enable the broiler industry to implement more effective *Salmonella* control strategies.

This study aimed to identify *Salmonella* serovar profiles at different stages of broiler live production to determine trends and potential serovar-mediated transmission dynamics. This was accomplished in two steps. First, to track broiler breeder flocks and their progeny, we collected environmental samples from six commercial complexes across four southeastern states, including samples from broiler breeders, hatcheries, and broiler houses both before and 7 days after chick placement. Second, positive samples were deep-serotyped using CRISPR-SeroSeq to determine *Salmonella* serovar populations and track their movement across different production stages within each complex.

## Materials and methods

### Sample collection

**Figure 1:**
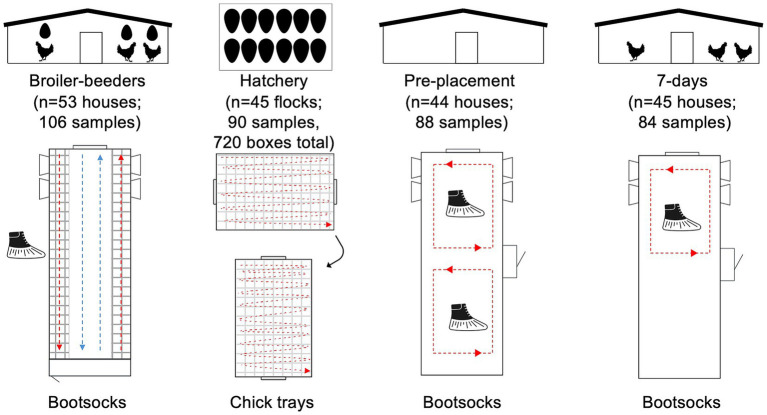
Study design. For breeder houses, bootsock samples were collected from the slats (red arrows) and the floor (blue arrows). For hatchery samples, 16 chick trays were swabbed (four trays per bootsock, two socks per sample) for each corresponding broiler house, as indicated by red arrows. Broiler houses at pre-placement and 7 days after placement were sampled using bootsocks. At 7 days, only the brood area was sampled.

Six integrated complexes (complexes 1–6) were sampled in spring 2025 across four different states. In total, three complexes (complexes 1, 4, and 5) were sampled twice, for a total of nine samplings (Table 1). As this was a paired study, samples were collected from broiler breeder houses, and the transmission of *Salmonella* to their offspring was tracked in the subsequent stages. Pre-moistened bootsock pairs (Romer Labs, DE) were used to sample the slats and floors of 53 breeder houses (*n* = 106 samples), and they were collected 2–3 days after the eggs were placed in the setter (Figure 1). On the day of placement, two types of samples were collected: First, two pairs of bootsocks were taken from each empty broiler house (“pre-placement”) (*n* = 44 houses); a pre-placement sample was not collected from one house in complex 3. Second, the samples were collected using bootsocks to wipe the inside of chick boxes after chick removal. Here, a pre-moistened bootsock was placed over a freshly gloved hand and used to wipe four chick boxes, as shown in Figure 1. In total, 16 boxes were sampled per destination broiler house (i.e., two pairs of bootsocks per house). For the first hatchery, this was performed at the hatchery, after allowing the chicks to sit in the basket for 2 h before the sample collection. For subsequent visits, 16 chick boxes per broiler house were marked with flagging tape. When placed on the farm, the boxes were not set directly on the floor. The boxes were then swabbed upon their return to the hatchery. In total, 90 bootsock pairs were used to collectively sample 720 chick boxes. Finally, samples were collected from the same broiler houses 7 days after placement, using two bootsock pairs that were sampled in the brood area only (*n* = 84). In complex 1, six houses had only a single pair of bootsocks collected. A detailed list of the samples collected in this study can be found in Supplementary Table 1. The samples were shipped on ice packs to the University of Georgia for analysis.

**Table 1:** Facilities sampled during the study.

Complex	Breeder	Chick trays	Pre-placement	Day-7
1^a^	7	8	8	8
2	2	5	5	5
3	8	6	5	6
4^a^	15	10	10	10
5^a^	13	12	12	12
6	8	4	4	4
Total	53	45	44	45

a Complexes were sampled twice during the study.

### Sample processing and prevalence

The sample culture began on the day the samples arrived in the laboratory. The samples were cultured following the National Poultry Improvement Plan (NPIP) protocol (36). Bootsocks from broiler breeder and 7-day broiler samples were enriched in 200 mL of tetrathionate (TT) broth (Hardy Diagnostics, Santa Maria, CA, USA) supplemented with 4 mL of iodine (Hardy Diagnostics, Santa Maria, CA, USA) and incubated at 37 °C for 24 h. The samples were directly transferred to selective media: Xylose-Lysine-Tergitol 4 (XLT4) (Hardy Diagnostics, Santa Maria, CA, USA) and Brilliant Green Sulfa agar supplemented with novobiocin (40 mg/L) (BGN) (Becton Dickinson, Sparks, MD, USA), and incubated at 37 °C for 24 h. In addition, 100 μL of TT broth was also transferred to Modified Semisolid Rappaport Vassiliadis (MSRV) agar supplemented with novobiocin (20 mg/L) (Becton Dickinson, Sparks, MD, USA) and incubated at 42 °C for 24 h. If there was a zone of growth on the MSRV agar, a single 10 μL inoculating loop was used to stab into the agar in three different locations within the external ring of growth, and the material was then streaked onto XLT4 and BGN agars as described above. Presumptive *Salmonella* colonies from XLT4 and BGN were transferred to Luria-Bertani (LB) agar (Fisher Scientific, Fair Lawn, NJ, USA) and incubated at 37 °C for 24 h. The isolates were confirmed by agglutination using *Salmonella* antiserum poly A-I & Vi (Becton Dickinson, Sparks, MD, USA).

As we expected a lower *Salmonella* load in the hatchery and pre-placement samples, these were cultured using an alternative NPIP protocol that uses a non-elective pre-enrichment to increase overall bacterial growth, including *Salmonella*, followed by selective enrichment in TT broth at a higher temperature. The samples were enriched in 200 mL of Buffered Peptone Water (BPW) (Hardy Diagnostics, Santa Maria, CA, USA) and incubated at 37 °C for 24 h. Then, 1 mL and 100 μL of BPW were transferred to 9 mL of TT broth and MSRV, respectively, and incubated at 42 °C for 24 h. Positive samples were plated on XLT4 and BGN and incubated at 37 °C for 24 h, and colonies were selected and confirmed as described above. As two pairs of bootsocks were collected for each of the four sample timepoints/locations (except for the six single 7-day samples from complex 1), the house or chick boxes were considered positive if at least one of the pairs tested positive.

### DNA extraction and CRISPR-SeroSeq

A total of 1.5 mL of the overnight TT culture was transferred to a microcentrifuge tube and centrifuged for 3 min at 18,000*g*. The supernatant was discarded, and the pellets were stored at −20 °C. For the *Salmonella-*positive samples, DNA was extracted from the pellets using the Promega Wizard Genomic kit following the manufacturer’s instructions (Promega, Madison, WI, USA). The DNA was resuspended in 200 μL of molecular-grade water and stored at −20 °C. CRISPR-SeroSeq libraries were generated using a single-step PCR, as previously described (37). In this approach, the primers target the conserved direct repeat sequences in the native CRISPR arrays of *Salmonella,* and the primer tails contain index sequences that facilitate sample multiplexing, followed by Illumina adaptor sequences. The samples were single-end sequenced on an Illumina NextSeq using 150 cycles. Serovar profiles were generated as previously described (37), and CRISPR reads for each sample were normalized using DeSeq2 (38). To generate a single *Salmonella* serovar profile from up to two samples collected from the same house (or chick box sets) on the same day, the samples were combined by averaging the relative serovar frequency from both CRISPR-SeroSeq libraries. For the samples where CRISPR-SeroSeq amplification failed (*n* = 17), isolated colonies (*n* = 26) were agglutinated with O antigen-specific sera, and the serovar was confirmed using serovar-specific qPCR assays (Table 2). For the samples where serovar Typhimurium was detected, the sample template DNA used for CRISPR-SeroSeq was used as a template for a PCR targeting a commercial serovar Typhimurium vaccine (39). The Illumina sequences generated from the CRISPR-SeroSeq amplicons are available in the Sequence Read Archive (SRA) at NCBI under BioProject PRJNA1424579.

**Table 2:** Primer sequences used for serovar confirmation by colony qPCR.

Serovar	Primer	Sequence (5′–3′)	Reference
Enteritidis	Forward	GCTGAAAACGGTTTTTCGGTCCG	This study
	Reverse	CGGGGAACACCATGGCAATT	
	Probe	CGGACGTGCTCGCTCTCGGTTTATCCC	
Kentucky	Forward	GATAAACCGCGCCCCTCAC	This study
	Reverse	CGCGGGGAACACAAATCGTT	
	Probe	CCGCGTTAAACATAGTTGCCGGTTTATCCCC	
Infantis-1	Forward	CGTTGATTTTAATGGCGGGCGAATTG	(59)
	Reverse	CGGGGAACACGCGGAGAATTAT	
	Probe	CGGAATGTTACAGGAAACGGTTTATCCCCGC	
Infantis-2	Forward	TCGTACACCAGCGCTTTACCG	(59)
	Reverse	CGGGGAACACGGTTTGCC	
	Probe	CAGCGGGGATAAACCGATCAAATATCAGATAACCC	
Mbandaka	Forward	ACCGGTACGGAAATTTGTGTCAGA	(60)
	Reverse	GGGAACACTATCCTGCGCAATTC	
	Probe	CGAACTGTGGGCACGGTTTATCCCC	

### Statistical analysis

Analysis was conducted using statistical software R (V4.4.0). Fisher’s exact test and the Kruskal–Wallis test, followed by Dunn’s *post hoc* test, were performed to evaluate differences in *Salmonella* prevalence and serovar complexity, respectively, across different production stages. The Shannon Diversity Index was used to assess serovar diversity at each stage, and the Kruskal–Wallis test was used to analyze sample richness across stages. This analysis was performed using the serovar profiles obtained from CRISPR-SeroSeq libraries.

## Results

### *Salmonella* prevalence across the production chain

**Figure 2:**
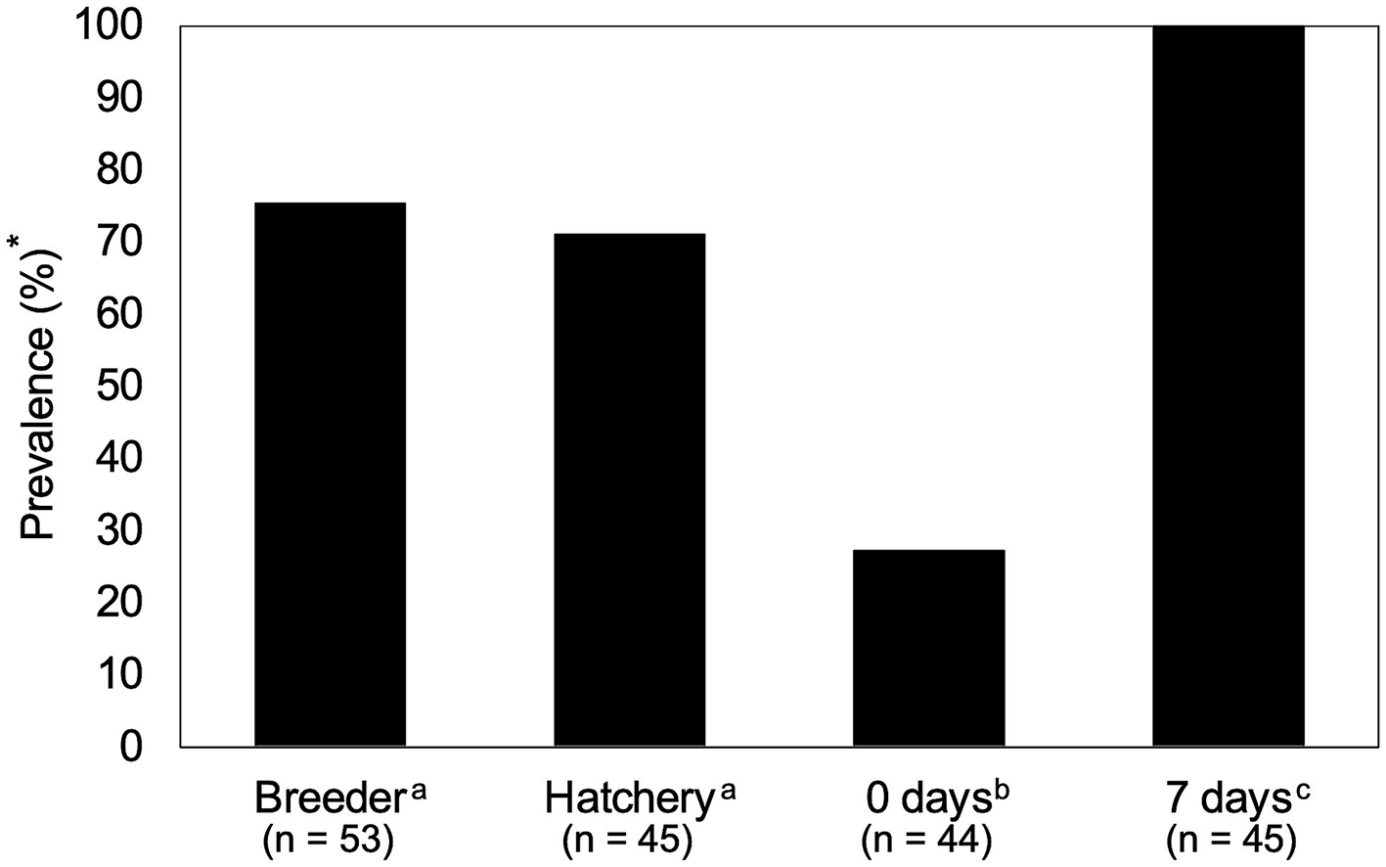
*Salmonella* prevalence during production stages. The bar graph shows *Salmonella* prevalence at four different stages of live poultry production. Each sample was collected in duplicate (e.g., right and left sides of the house), and a sample was considered positive if at least one sample of the two replicates tested positive.

To assess *Salmonella* in live broiler production, the samples were collected from broiler breeder flocks (*n* = 53) and flock-matched chicks via sampling of chick boxes at the hatchery (*n* = 45 samples across 720 chick boxes), as well as from broiler farms pre- and post-placement (Figure 1). A total of three-quarters (40/53) of broiler breeder houses were *Salmonella-*positive, with comparable prevalence in chick trays at 71.1% (32/45) (Fisher’s exact test, *p* > 0.05). The lowest *Salmonella* recovery was observed in pre-placement samples, with a prevalence of 27.3% (12/44). Finally, after 7 days of placement, all houses were *Salmonella-*positive (100%; 45/45). Fisher’s exact test analysis determined that both pre-placement and 7-day samples differed significantly from the other samples (Figure 2; *p* < 0.05). Therefore, these prevalence data indicate that broiler breeder flocks are a significant source of *Salmonella* in broiler houses.

### *Salmonella* serovar complexity

**Figure 3:**
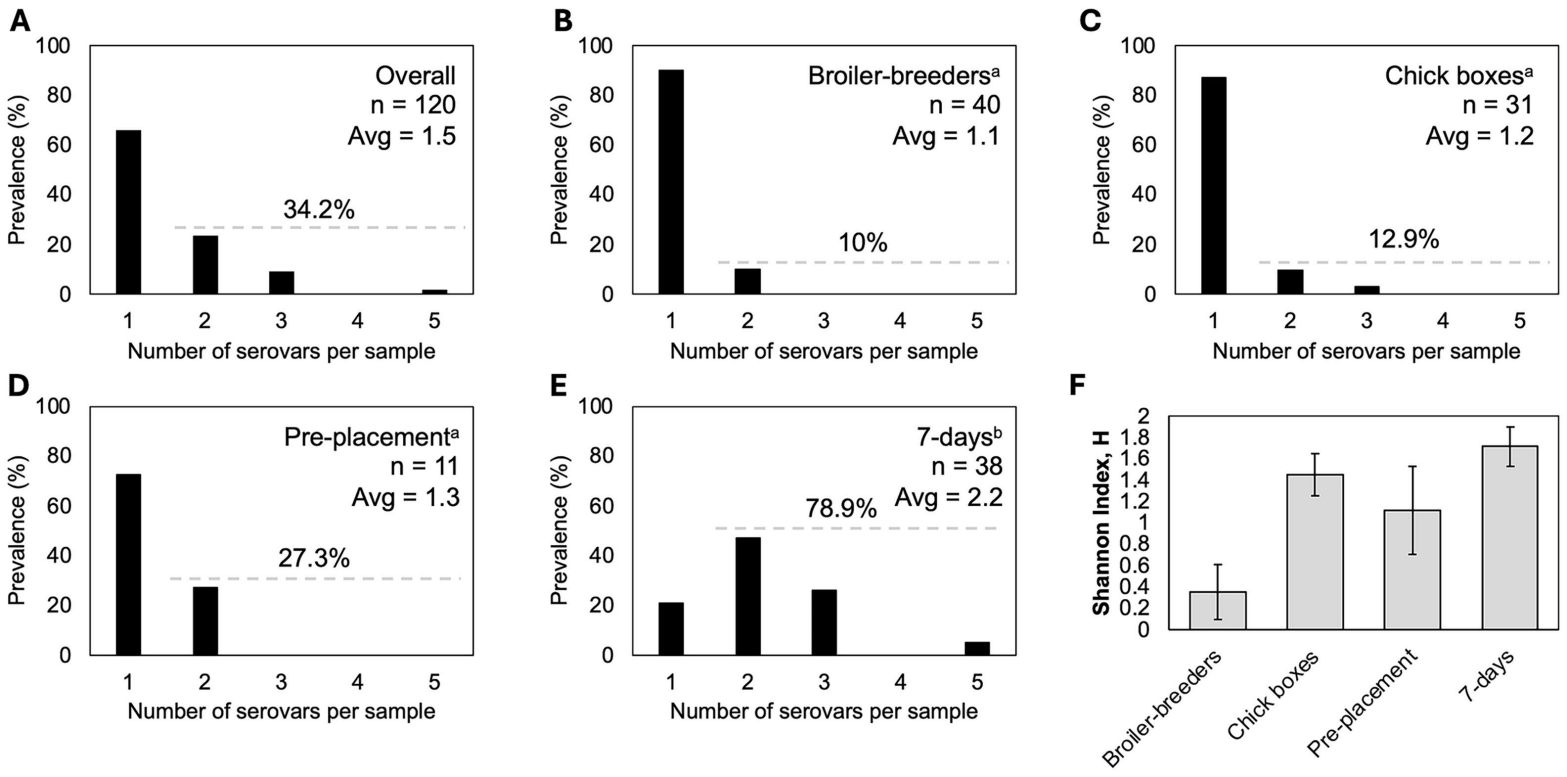
Serovar complexity analysis. **(A)** Overall prevalence of multi-serovar populations across the study assessed using deep serotyping data (*n* = 120). **(B–E)**. Serovar complexity by production stage. Statistical differences determined using the Kruskal–Wallis test (*p* < 0.01), followed by Dunn’s *post hoc* test, are indicated by superscript letters. **(F)** Serovar diversity across production stages, measured using the Shannon Diversity Index.

**Figure 4:**
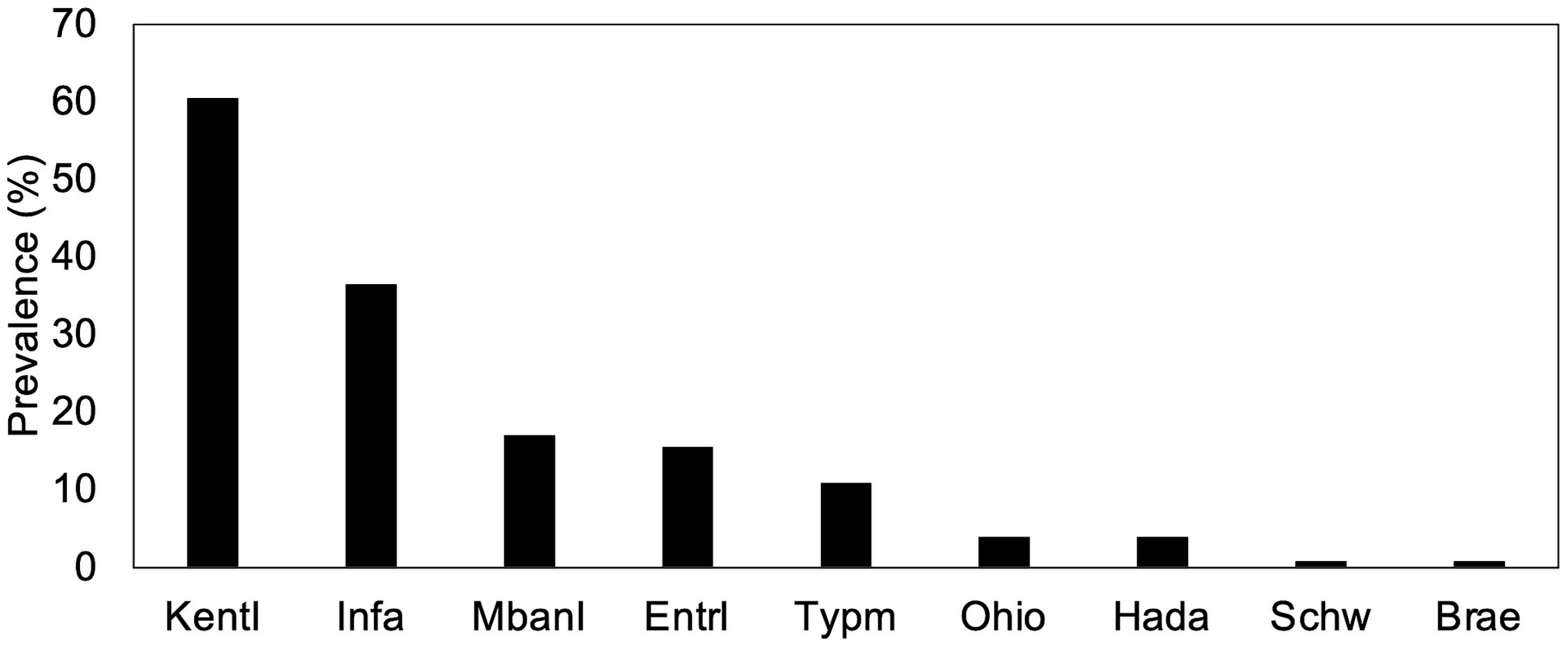
Serovar frequencies. The frequencies of individual serovars are shown. Serovars from left to right: KentI, Kentucky; Infa, Infantis; Mban, Mbandaka; Entr, Enteritidis; Typm, Typhimurium; Hada, Hadar; Ohio, Ohio; Schw, Schwarzengrund; Brae, Braenderup.

To determine *Salmonella* serovar profiles, deep serotyping by CRISPR-SeroSeq was performed on all *Salmonella*-positive samples (231/368), and usable libraries were generated for 214 samples. To analyze serovar complexity per individual broiler breeder house, broiler house, and set of chick boxes, the results from the samples collected from the same house or set of chick boxes were combined into a single profile, as previously demonstrated to capture the full serovar composition within a house (35). After combining the two samples per house or per set of 16 chick boxes, a single serovar profile was generated for each house or chick box set. Subsequent references to ‘samples’ in this manuscript refer to these combined profiles (*n* = 120). Across all samples, nearly one-third (34.2%; 41/120 samples) contained more than one serovar, with an average of 1.5 serovars per sample (Figure 3). There was no significant difference in serovar complexity between breeder, hatchery, and pre-placement samples, but the 7-day samples exhibited significantly higher complexity, with an average of 2.2 serovars/sample (range, 1–5) and 78.9% of samples containing mixed serovars (Kruskal–Wallis, *p* < 0.01). Total serovar diversity was lowest in broiler breeder samples (*n* = 3 serovars) and highest in 7-day broiler house samples (*n* = 9 serovars), as shown by the Shannon Diversity Index values (Figure 3F). A total of nine different serovars were detected across the study (Figure 4). As expected, serovar Kentucky was the most prevalent (60.5%, 78/129 samples), including isolates that were serotyped when deep serotyping failed. Serovars Infantis (36.4%, 47/129), Mbandaka (17.1%, 22/129), and Enteritidis (15.5%, 20/129) were also frequently detected.

### Transmission patterns of individual serovars

**Figure 5:**
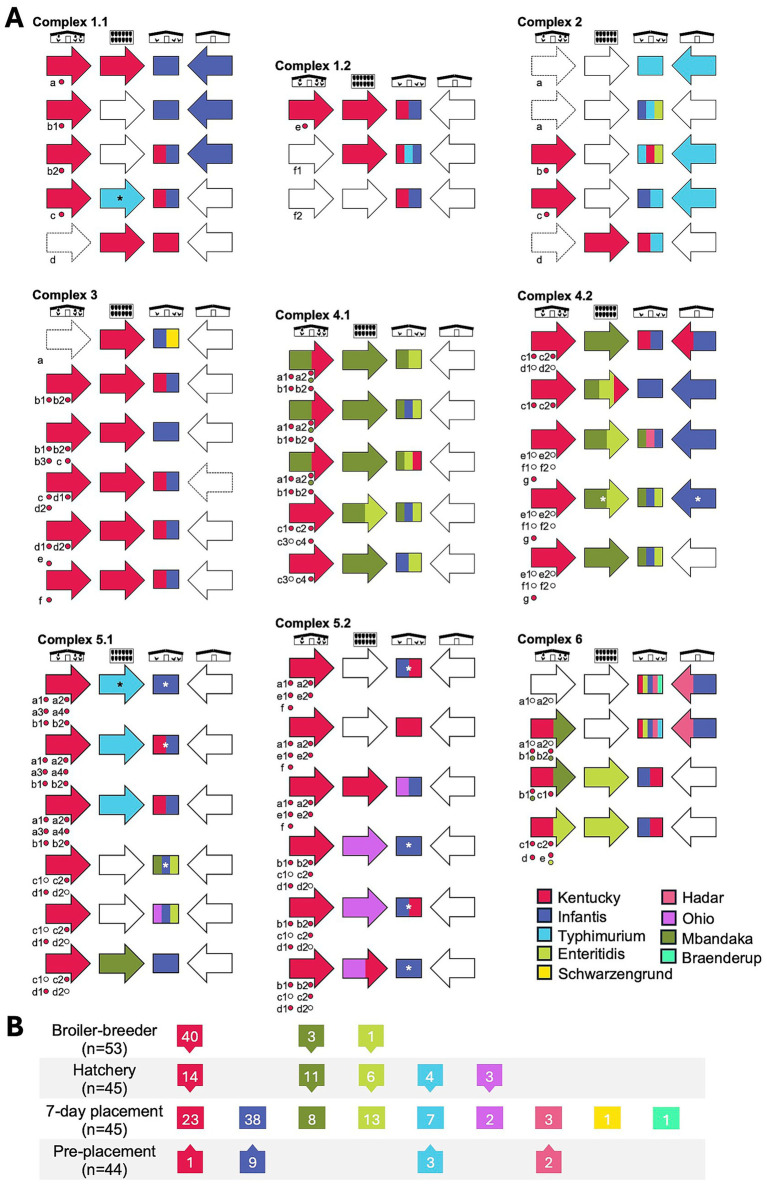
Serovar patterns traced across live poultry production stages in six complexes. **(A)** Complexes 1, 4, and 5 were visited twice. Black and white symbols at the top of each complex represent, from left to right, broiler breeder, hatchery, 7-day, and pre-placement samples. Filled arrows and boxes depict *Salmonella* serovar(s) present, as indicated in the key. White boxes indicate *Salmonella*-negative samples. In most cases, broiler chicks placed in a single house originated from multiple broiler breeder farms or houses. To capture this, lowercase letters indicate the identity of a broiler breeder farm within a complex, and the number represents the house. During a single visit to a complex, these letters indicate chicks from broiler breeder flocks that were placed in multiple broiler houses. A white asterisk indicates that serotype information was derived from colonies where CRISPR-SeroSeq libraries failed. A black asterisk indicates that the detected serovar Typhimurium was a commercial vaccine strain. Dashed outlines on arrows indicate that a sample was not collected. **(B)** Serovar frequency by production stage.

We next sought to determine whether there were differences in serovar distributions at different stages of production, as these might indicate enriched transmission patterns for some serovars. Serovar Kentucky was found in broiler breeder flocks from all complexes, in all six hatcheries (except during the first visits to complexes 4 and 5; 4.1 and 5.1), and in broiler farms from all complexes at 7 days post-placement (Figure 5A). Interestingly, at pre-placement, serovar Kentucky was only detected in one complex (complex 4.2). This suggests that serovar Kentucky present upstream in breeders is responsible for colonizing broiler flocks. However, despite its pervasiveness in breeders (40/53 flocks, including 36 where it was the only serovar) and hatcheries (14/45 sets of chick box samples), serovar Kentucky was only detected in 23/45 broiler houses 7 days after placement (Figure 5B). Similarly, serovars Enteritidis and Mbandaka were absent in pre-placement broiler houses, and their prevalence was higher in hatcheries than in breeders. Serovar Enteritidis was detected once in breeders (complex 6) and three times in hatcheries (complexes 4.1, 4.2, and 6). On broiler farms, it was detected in 13 houses across four complexes. Serovar Mbandaka had similar patterns and was almost exclusive to complex 4. Although less prevalent, serovar Ohio was exclusive to complex 5 and was only detected in chick box swabs (*n* = 3) and in two houses 7 days post placement. Serovar patterns are summarized in Figure 5B.

Serovar Infantis was the second most frequently detected serovar in this study, present in 36% of samples (Figure 4). It was exclusively found in pre-placement and 7-day post-placement samples and was never observed in broiler breeder or hatchery samples, despite the higher depth of serotyping provided by CRISPR-SeroSeq. At 7 days post-placement, serovar Infantis was found in all complexes, in all but four houses, and was the most frequently detected serovar in pre-placement samples (75%; 9/12 positive pre-placement houses) (Figure 5). This suggests that broilers are colonized with residual serovar Infantis present in the house from the previous flock. Serovar Typhimurium was detected less frequently and was found in chick boxes, pre-placement samples, and 7-day samples. For two hatchery samples (one in complex 1 and one in complex 5), the strain of serovar Typhimurium was determined to be the live-attenuated vaccine strain by PCR. Finally, four other serovars (serovars Ohio, Hadar, Schwarzengrund, and Braenderup) were found but were infrequent enough to prevent us from assessing any transmission patterns. Collectively, these data highlight differences in serovar prevalence across the different stages of broiler production. These differences subsequently influence how broiler flocks become colonized with different *Salmonella* serotypes and highlight the importance of not relying only on *Salmonella* prevalence data.

## Discussion

*Salmonella* remains a significant challenge for broiler producers, and effective control depends on understanding its transmission within vertically integrated poultry production systems. *Salmonella* is not a one-size-fits-all pathogen. Due to their differing abilities to colonize various animals, survive on different food products, and ultimately cause human illness, different serovars (and their subtypes) can have vastly different impacts on public health (5, 13, 40). Therefore, to understand *Salmonella* dynamics during broiler production, it is imperative to consider how different serovars are transmitted.

In this study, *Salmonella* prevalence and potential serovar transmission patterns were assessed. We found that prevalence in broiler breeders exceeded 70%, which is higher than reported in other studies, where prevalence ranged from 5 to 35% (17, 33, 41). Although studies on broiler breeders are much fewer than those on broilers, this finding may reflect variability across individual operations. For example, a recent study showed that *Salmonella* prevalence in broiler breeder flocks was 35% (1,581/4,485 samples), with samples collected from birds of multiple ages across more than 20 companies. In the same study, 13 individual broiler breeder flocks from just two commercial complexes were monitored over time, and *Salmonella* prevalence ranged from 62 to 92%, depending on flock age, peaking as hens were coming out of peak production at 38 weeks (17). Prevalence in pre-placement samples was 27.3% in this study, which is comparable to the findings of Kim et al. (42), who reported that prevalence in dust samples varied from 0 to 25% among sampled farms. Moreover, other studies have reported a prevalence of 29% in pre-placement samples (43, 44). Finally, *Salmonella* prevalence at 7 days was highest in this study, with *Salmonella* present in all sampled houses. This is comparable to other studies that used environmental bootsock sampling to assess *Salmonella,* which reported 44–97% of samples as positive (35, 45–47). Based on prevalence alone, these data suggest that the main route for *Salmonella* colonization of broilers is via broiler breeders, a conclusion supported by a recent meta-analysis indicating that hatcheries are a significant contributor to *Salmonella* transmission (48).

Resolution to the serovar level, however, tells a very different story. At the serovar level, we see notable differences in serovar transmission. To assess overall serovar profiles per sampling unit (house or chick box), two samples collected from the same house or chick box were combined into one. Broiler breeders seem to be the primary transmission source of *Salmonella* serovars Kentucky, Enteritidis, and Mbandaka. Here, we found that serovar Kentucky was the most prevalent, identified in more than 60% of sampled facilities and present at all four sampling stages. Serovar Kentucky was consistently the most frequently identified across all stages of broiler production, reflecting its strong adaptation to poultry, including multiple stages of live bird production and processing plants (11, 13, 17, 35, 40, 49–53). Analysis of serovar co-occurrence in 568 broiler breeder flocks showed that serovar Kentucky competitively excludes other serovars (17), a phenomenon attributed to its higher expression of *rpoS*-regulated genes compared to serovar Typhimurium when grown in a chicken cecal contents (50). Therefore, it was somewhat unexpected that serovar Kentucky was only found in two 7-day-old broiler houses as the only serovar. It is possible that, had we continued sampling the broiler houses, serovar Kentucky would have continued to expand, potentially outcompeting other serovars. Alternatively, the reduced prevalence of other *Salmonella* serovars in broiler breeders may be explained by the fact that some serovars are suppressed in broiler breeders due to the application of live-attenuated and killed vaccines (33, 54).

Prevalence patterns of serovars Enteritidis and Mbandaka also suggest transmission from broiler breeders to broilers. True vertical transmission occurs when the ovaries are colonized by *Salmonella,* allowing the bacteria to pass into the egg and the chick after hatching (18). Conversely, pseudo-vertical transmission occurs when *Salmonella* contaminates the eggshell, which the chick may ingest while pipping (23, 24). A recent study showed that a serovar Enteritidis isolate collected from a breeder sample differed by fewer than 14 SNPs from an isolate from a broiler farm, while multiple isolates from hatcheries differed by fewer than 6 SNPs from isolates from broiler houses, suggesting that closely related isolates move across production stages (49). Similarly, another study found that the same subtypes identified in the hatchery were also present in broiler houses (20). Multiple studies have demonstrated true vertical transmission of serovar Enteritidis in chickens (18, 55, 56), which is due to the unique ability of this particular serovar to systemically infect chickens (i.e., colonize tissues outside of the GI tract). This is also supported by USDA-FSIS data showing a significantly higher proportion of serovar Enteritidis in broiler parts than in carcasses (11). Therefore, given that serovar Enteritidis can colonize other tissues or organs, its low prevalence in environmental samples from breeder flocks may reflect its absence from the GI tract and subsequent lack of shedding. In our study, serovar Mbandaka was detected in broiler breeders and/or hatcheries from three different complexes, which is consistent with the relatively frequent incidence of this serovar reported in another study of broiler breeders (17). However, this serovar is most often associated with beef cattle and is rarely detected by the USDA-FSIS in poultry products (9). Therefore, it was unexpected to see it present in broiler flocks. Our previous publication hypothesized that the suppression of serovars of concern through vaccination may allow non-target serovars to colonize chickens (17). Then, without vaccine pressure in broilers, these serovars are less competitive than those better adapted to poultry. Unfortunately, our study ended at 7 days; it would have been interesting to see whether serovar Mbandaka persisted throughout production. We suspect that both serovars Mbandaka and Enteritidis are pseudo-vertically transmitted; for the latter, it could also be vertically transmitted, although the frequency is typically low (55, 57).

Serovar Infantis was only detected in broiler houses and never upstream. This is a strong indication that it persists in broiler house environments. This is further supported by its high prevalence in pre-placement samples, where it was the most frequent serovar (75%; 9/12). Given that three-quarters of 7-day samples (34 of 45 houses) contained multiple serovars (average 2.1 serovars per house), it is reasonable to assume that the previous flocks in these houses also had multiple serovars. Therefore, it was somewhat unexpected that, when *Salmonella* was recovered at pre-placement, the prevalence was low (12/44 houses), and only a quarter of houses contained multiple serovars. This suggests that house management practices between flocks reduced both *Salmonella* prevalence and complexity. It is interesting that, despite the presence of nine different serovars in 7-day houses, serovar Infantis was detected in 84% of houses and accounted for 75% of pre-placement samples. Collectively, these findings suggest some kind of selection for serovar Infantis on the farm, perhaps due to litter management during flock downtime. Given its pervasiveness in poultry, it was unexpected that serovar Kentucky was not frequently detected in pre-placement samples. This suggests that whatever the management methods are, Kentucky is more susceptible to control measures. A study showed that *Salmonella* could be isolated from deep soil in a broiler house (58); therefore, the difference in prevalence between serovars Kentucky and Infantis may reflect differences in environmental fitness and subsequent persistence, which should be explored further.

As a caveat, the serovar dynamics presented here are based on serotyping data, which limits the resolution for assessing clonal links between different production stages. Future experiments could incorporate molecular subtyping or WGS approaches to confirm these connections between production stages. Furthermore, *Salmonella* shedding may vary over time in birds, potentially resulting in differences at the serovar level. Future experiments that track *Salmonella* serovar populations throughout grow-out phases and into processing will provide additional context to the findings presented here.

Taken together, the findings of this study indicate that *Salmonella* is well adapted to multiple stages of broiler production. Notably, certain serovars exhibit distinct transmission patterns. This has important implications for poultry producers, suggesting that effective control strategies should be tailored to the specific serovars present.

## Data Availability

The datasets presented in this study can be found in online repositories. The names of the repository/repositories and accession number(s) can be found at: https://www.ncbi.nlm.nih.gov/, PRJNA1424579.
